# Impact of Intensive Gait Training With and Without Electromechanical Assistance in the Chronic Phase After Stroke–A Multi-Arm Randomized Controlled Trial With a 6 and 12 Months Follow Up

**DOI:** 10.3389/fnins.2021.660726

**Published:** 2021-04-22

**Authors:** Susanne Palmcrantz, Anneli Wall, Katarina Skough Vreede, Påvel Lindberg, Anna Danielsson, Katharina S. Sunnerhagen, Charlotte K. Häger, Jörgen Borg

**Affiliations:** ^1^Department of Clinical Sciences, Danderyd Hospital, Karolinska Institutet, Stockholm, Sweden; ^2^Institut de Psychiatrie et Neurosciences de Paris, Inserm U1266, Université de Paris, Paris, France; ^3^Institute of Neuroscience and Physiology, Rehabilitation Medicine, University of Gothenburg, Gothenburg, Sweden; ^4^Department of Health and Rehabilitation, Institute of Neuroscience and Physiology, University of Gothenburg, Gothenburg, Sweden; ^5^Section for Physiotherapy, Department of Community Medicine and Rehabilitation, Umeå University, Umeå, Sweden

**Keywords:** stroke rehabilitation, robotics, ambulation, walking, treadmill

## Abstract

**Introduction:** Movement related impairments and limitations in walking are common long-term after stroke. This multi-arm randomized controlled trial explored the impact of training with an electromechanically assisted gait training (EAGT) system, i.e., the Hybrid Assistive Limb^®^ (HAL), when integrated with conventional rehabilitation focused on gait and mobility.

**Material and Methods:** Participants, aged 18–70 years with lower extremity paresis but able to walk with manual support or supervision 1–10 years after stroke, were randomized to (A) HAL-training on a treadmill, combined with conventional rehabilitation interventions (HAL-group), or (B) conventional rehabilitation interventions only (Conventional group), 3 days/week for 6 weeks, or (C) no intervention (Control group). Participants in the Control group were interviewed weekly regarding their scheduled training. Primary outcome was endurance in walking quantified by the 6 Minute Walk Test (6MWT). A rater blinded to treatment allocation performed assessments pre- and post-intervention and at follow-ups at 6 and 12 months. Baseline assessment included the National Institute of Health Stroke Scale (NIHSS) and the Modified Ranking Scale (MRS). Secondary outcomes included the Fugl Meyer Assessment- Lower Extremity, 10 Meter Walk Test, Berg Balance Scale (BBS), Barthel Index (BI) and perceived mobility with the Stroke Impact Scale.

**Results:** A total of 48 participants completed the intervention period. The HAL-group walked twice as far as the Conventional group during the intervention. Post-intervention, both groups exhibited improved 6 MWT results, while the Control group had declined. A significant improvement was only found in the Conventional group and when compared to the Control group (Tukey HSD *p* = 0.022), and not between the HAL group and Conventional group (Tukey HSD *p* = 0.258) or the HAL- group and the Control group (Tukey HSD *p* = 0.447). There was also a significant decline in the Conventional group from post-intervention to 6 months follow up (*p* = 0.043). The best fitting model to predict outcome included initial balance (BBS), followed by stroke severity (NIHSS), and dependence in activity and participation (BI and MRS).

**Conclusion:** Intensive conventional gait training induced significant improvements long-term after stroke while integrating treadmill based EAGT had no additional value in this study sample. The results may support cost effective evidence-based interventions for gait training long-term after stroke and further development of EAGT.

**Trial registration:** Published on clinicaltrials.gov (NCT02545088) August 24, 2015.

## Introduction

Stroke is one of the most common causes of acquired adult disability world-wide (Feigin et al., 2018; Kim et al., 2020). The effects of stroke are complex but are frequently manifested as a hemiparesis that impacts on gait function (Jorgensen et al., 1995). Depending on location, the extent of the lesion and of restorative and compensatory mechanisms, gait characteristics may vary between patients and over time after stroke. Although early rehabilitation interventions after stroke target independence in mobility and activities of daily living, self-perceived limitations in these areas commonly remain (Alguren et al., 2010) and around one third have reported need of assistance in walking long-term after stroke (Jorgensen et al., 1995; The Swedish Stroke Register Riksstroke, 2018). Thus, limitations in walking is still a challenge long-term after stroke.

Within rehabilitation, conventional gait training after stroke may include over ground walking with assistance and/or ambulatory devices or walking on a treadmill, with or without body weight support (BWS). Conventional gait training can be combined with electromechanically assisted gait training (EAGT), which may allow more reproducible gait movements than manual movement support by therapists.

Currently, there are a number of exoskeletons with different clinical applications. These devices differ in mechanical design and control strategies as well as in how they facilitate movement of the limb (Chen et al., 2013; Sanchez-Villamanan et al., 2019). Recent reviews on the effect of using EAGT after stroke report contradictive results. While e. g., Bruni et al. (2018) found additional improvements in gait speed compared to conventional training in the subacute phase after stroke, Tedla et al. (2019) found no additional effect on gait speed in the subacute phase but a positive trend in the chronic phase. A Cochrane review by Mehrholz et al. (2017b) found no additional effect on gait speed irrespective of time after stroke. Nevertheless, additional improvements in independence in walking was found in the subacute phase (Mehrholz et al., 2017b). To fully evaluate potential benefits of using current types of EAGT to improve walking and ambulation, future research should focus on individual characteristics impacting on the outcome and blinded randomized control trials with long-term follow ups (Mehrholz et al., 2017b; Bruni et al., 2018; Molteni et al., 2018; Tedla et al., 2019).

**FIGURE 1:**
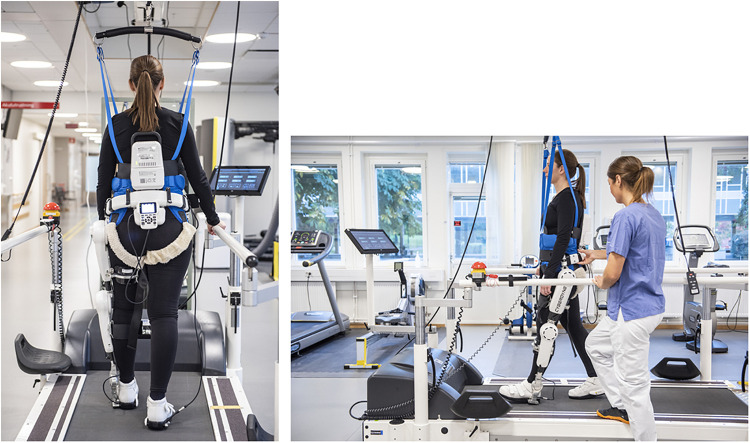
HAL used on a treadmill with body weight support (BWS). Consent for publication was obtained from the persons in the pictures (photo: Johan Adelgren).

Over the last few decades an exoskeleton with a hybrid system that allows both an automatic and a voluntary mode of action to support gait movements and training of gait, the Hybrid Assistive Limb^®^ (HAL; Cyberdyne, Tsukuba, Japan) has been developed, introduced and tested in clinical trials (Kawamoto et al., 2010; Wall et al., 2015; Sczesny-Kaiser et al., 2019; Wall et al., 2020). This exoskeleton provides support according to the patient’s condition by a control algorithm and supporting devices, where each joint (left and right hip and/or left and right knee) can be controlled separately by the therapist (Figure 1). The HAL system has been described in detail previously (Kawamoto and Sankai, 2002; Suzuki et al., 2007; Kawamoto et al., 2010).

The system comprises two subsystems for voluntary control (CVC) and autonomous control (CAC), respectively. The CAC mode utilizes voluntary weight shift to initiate gait cycles and then provides predefined movements while gait in the CVC mode continuously uses input from voluntarily activated gait muscles to provide support by the exoskeleton. This is achieved by use of surface electromyography (EMG) signals from lower extremity extensor and flexor muscles to initiate and adapt power output, which may then be modified by a therapist. The technology enables even weak EMG activity to be used to initiate and adjust the assistive torque in the CVC mode.

The feasibility, safety and potential functional benefits of gait training with HAL after stroke have been demonstrated (Kawamoto et al., 2013; Nilsson et al., 2014; Watanabe et al., 2014). Consequently, a review (Wall et al., 2015) identified consistent evidence that the use of the HAL system is feasible and safe when used for gait training in hospital and rehabilitation settings early and late post-stroke. In a recent RCT (Wall et al., 2020), in a younger study population (median 55 and 57.5 years) with severe limitations in walking in the subacute stage after stroke, we explored the effects of training with HAL combined with conventional rehabilitation interventions and compared to conventional rehabilitation interventions only. Results showed large improvements in sensorimotor function, activity and independence in walking but with no significant between group differences (Wall et al., 2020).

In the long-term phase after stroke, reports from Japan indicate that training with HAL may improve walking (Nankaku et al., 2020) also when compared to conventional training (Yoshimoto et al., 2015). However, these results needed to be confirmed in a controlled study and in comparison with conventional evidence-based rehabilitation interventions (Mehrholz et al., 2017b; Bruni et al., 2018; Molteni et al., 2018; Tedla et al., 2019).

Thus, the overall aim of this study was to explore potential additional effects on functioning of training with the Hybrid Assistive Limb (HAL), when integrated with conventional rehabilitation focused on gait and mobility in the long-term phase (1–10 years) after stroke. Specific aims were to explore (1) if a matched training period using the HAL method or a conventional method improved impaired sensorimotor function, balance and walking ability and (2) if there were differences between intervention groups or (3) compared to a control group and (4) if these potential effects within training groups and differences between training groups changed over time. Additional aims were to explore (5) factors affecting outcome such as patient characteristics and initial level of functioning and (6) if these factors differed between groups and (7) over time.

## Materials and Methods

### Design and Setting

The study is a randomized controlled trial conducted at the University Department of Danderyd Hospital and Department of Clinical Sciences at Karolinska Institutet in Stockholm. The study protocol has been approved by the Swedish Ethical Review Authority (Dnr: 2015/1216-31) and published on clinicaltrials.gov (NCT02545088). Study design and manuscript follow the extension of the CONSORT 2010 statement for multi-arm parallel group randomized trials and are in accordance with the principles of the Declaration of Helsinki and Good Clinical Practice.

### Recruitment of Participants

Eligible were persons, aged 18–70 years, who had received conventional interventions due to stroke related hemiparesis of the lower extremity (LE) after an ischemic or hemorrhagic first ever stroke (diagnosed by a stroke physician and verified by CT or MRI). Participants were recruited from out-patient rehabilitation units and through advertising. At the rehabilitation units, physiotherapists were informed about the study by the study coordinator. Eligible participants were reported to the study coordinator over the telephone, by the physiotherapist at the units, only after given consent from eligible participants. Thereafter, eligible participants were given verbal and written information about the study by the study coordinator, before giving informed consent to participate in the study.

#### Inclusion Criteria

1–10 years since stroke onset, able to walk but in need of manual support or close supervision due to lower extremity paresis i.e., a Functional Ambulatory Categories (FAC) score of 2–3 or ability to walk independently on even surfaces only i.e., FAC 4 in combination with reduced gait speed (<0.8 m/s according to 10 meter walk test), which corresponds to limitations in community ambulation (Holden et al., 1984; Perry et al., 1995; Bowden et al., 2008) ability to understand training instructions as well as written and oral study information and to express informed consent or by proxy; body size compatible with the HAL suit (height: ≥ 150 cm, weight: ≤ 100 kg).

#### Exclusion Criteria

Contracture restricting gait movements at any lower limb joint; cardiovascular or other somatic condition incompatible with intensive gait training; notifiable infectious disease, contagious infections.

### Randomization

Randomization was performed by a person, not otherwise involved in the study, according to a block design procedure where participants were randomized to (A) HAL-training combined with conventional rehabilitation intervention, or (B) conventional rehabilitation intervention without HAL or (C) no intervention. Randomization was performed after the first clinical baseline assessment of functioning (M1).

### Interventions

#### Group A: The HAL-Group

To standardize the training procedure, training with HAL was performed using BWS on a treadmill to enable safe gait training and unburden the weight of the suit (the BWS was pre-set to unburden 9 kg of the weight). The HAL suit is limited to motions in the sagittal plane, were assistance is given in hip flexion/extension and knee flexion/extension and the patients were instructed to avoid other (compensatory) movements (Suzuki et al., 2007; Chen et al., 2016). Training with HAL was performed 1 session/day, 3 days/week for 6 weeks and each session was not to exceed 1 h and 30 min (including approximately 30 min of donning and doffing of the exoskeleton and BWS). In addition, each session included conventional gait and mobility training limited to 30 min of training time. The training program was performed by one physiotherapist, trained in the HAL method and the study procedures, and an assisting physiotherapist when needed.

HAL training settings were individualized for each participant. The first session was performed by use of the CAC mode to allow time for initial adjustment of the suit (length and width) and to allow the patient to get acquainted with the suit within the set time period. In the following session adhesive electrodes were placed over the flexors and extensors at the knee and hip joint and the CVC mode was used. The settings of the exoskeleton were adjusted in order to achieve an optimal gait pattern as close to normal as possible, evaluated trough continuous clinical gait analysis (Kirtley, 2005) performed by the therapist during training. As the participants improved in walking ability, the amount of assistance given by the exoskeleton was reduced and walking speed on the treadmill was increased.

#### Group B: The Conventional Group

The conventional group received conventional gait and mobility training performed 1 session/day, 3 days/week for 6 weeks, not exceeding 1 h and 30 min/session (to match the scheduled training time in Group A). The conventional training was performed according to current practice in out-patient care in Sweden, and included over ground walking with assistance and/or assistant devices as well as the use of a treadmill without BWS and training of gait function, strength and balance.

#### Groups A and B

In both groups, at the end of the training period, the physiotherapist engaged in the participant’s conventional training, performed one home visit to inform/educate the participant and those who are providing assistance to the participant in how the participant could make use of any gains in gait function during activities of daily living.

#### Group C: The Control Group

The Control group did not receive any intervention but followed the same assessment protocol and were instructed to continue their every-day life activities as usual. The Control group was included to capture potential changes in functioning during the normal course of time when no specific training intervention was offered.

### Assessments

Assessments were made with standardized assessment tools and performed once at baseline (M1), immediately after the intervention period (M2) and at 6 (M3) and 12 (M4) months after the intervention period by a physiotherapist experienced in stroke rehabilitation who was blinded to the participants′ group allocation.

#### Screening of Functioning and Disability

Data on stroke type and localization were collected from medical records. Body function was assessed at M1 by use of the NIH Stroke Scale (ranging from 0 to 44 points, a higher score indicating more severe disability) (NIHSS) (Lyden et al., 1994). Independence in walking with the Functional Ambulation Categories [ranging from 0 (non-functional gait) to 5 (independent in walking)] (Holden et al., 1984) and activity and participation by means of the Modified Rankin Scale [ranging from 0 (no symptoms) to 6 (dead)] (Rankin, 1957; van Swieten et al., 1988).

#### Primary Outcome Measure

The 6 Minute Walk Test (6MWT) (Kosak and Smith, 2005) was used to assess endurance in walking by measuring distance accomplished during a 6 min walk. A turning point at 150 m was set for the first included participants in each group. Due to construction work, the turning point had to be shortened down to 100 m for the following nine participants and 50 m for the remaining participants.

#### Secondary Outcome Measures

Assessment of sensory function, pain, range of motion, and control of voluntary movements was performed with the Fugl Meyer Assessment- Lower Extremity (FMA-LE) (the motor function domain ranging from 0–34 points and the total score from 0 to 86 points). A lower score indicates more severe impairment (Fugl-Meyer et al., 1975).

Walking speed was tested with the 10 meter walk test and presented in meter/second (Wade et al., 1987) and the Borg RPE scale (Borg, 1982) for perceived exertion [ranging from 6 (no exertion) to 20 (maximal exertion)] was rated by the participants after the 6 MWT.

Balance in activity was assessed with the Berg Balance Scale (ranging from 0 to 56 points, a lower score indicating more limitations) (Berg et al., 1995), self-rated independence in mobility and personal care with the Barthel Index [ranging from 0 (fully dependent) to 100 points (independent)] (Mahoney and Barthel, 1965) and self-rated functioning and disability with the Stroke Impact Scale [each domain ranging from 0 (maximal limitation) to 100 points (no limitation)] (Duncan et al., 1999, 2003).

### Additional Data Collection

Conventional gait and mobility training in Group A and B was documented according to standardized protocols. The participants in the Control group (Group C), who did not receive an intervention, were interviewed regarding any scheduled training outside the study, in a brief structured telephone interview, once a week, according to a standardized protocol.

### Power Calculation and Statistics

Power calculation was based on a previous study on perceived and measured change in walking in the long-term phase after stroke (Tang et al., 2012). Using the 6 MWT as the primary outcome measure, a 34.4 m difference, a significance level of 5% and a power of 80%, the study required a minimum of 48 (16 × 3) participants in total.

Descriptive statistics are presented as mean and standard deviation (SD) for normally distributed continuous data and as median and interquartile range (IQR) for ordinal and not normally distributed data (detected with the Shapiro-Wilk test). Within group differences between two time points was assessed with Paired sample *t*-test [sig. (2-tailed) for normally distributed data and Related Samples Wilcoxon signed rank test for ordinal or not normally distributed data].

Between group differences were assessed with Independent Samples Test (normally distributed) or Mann Whitney *U* test (ordinal or not normally distributed data). Between group differences were assessed with one way ANOVA (*Post-hoc* test including Tukey HSD and Bonferroni) for normally distributed data or Independent samples Kruskal-Wallis test (ordinal or not normally distributed data).

Correlations were assessed using Pearson correlation for normally distributed data.

Linear regression was used to explore the association between dependent perceived improvements in mobility (Δ M1 M2, SIS Mobility domain) and independent factors related to changes (Δ M1 M2) in walking distance (6 minute walk test).

Linear Mixed Model for Longitudinal Data was applied to explore longitudinal changes in outcome variables. An unstructured repeated covariance structure was used. *Post-hoc* tests were adjusted according to Bonferroni. Dependent variable was the primary outcome, the 6 minute walk test, controlling for group allocation, the 4 time points (M1-M4) and interaction between group and the 4 time points. Predicting variables collected at M1 (Age, Sex, Diagnosis, Time to inclusion, Affected side, NIHSS, FMA-LE Berg Balance scale, Barthel Index and Modified Ranking Scale) were included in the model one at a time. Results from the significant and best fitting model with the smallest Akaike Information Criterion (AIC) were identified.

## Results

### Data Collection

**FIGURE 2:**
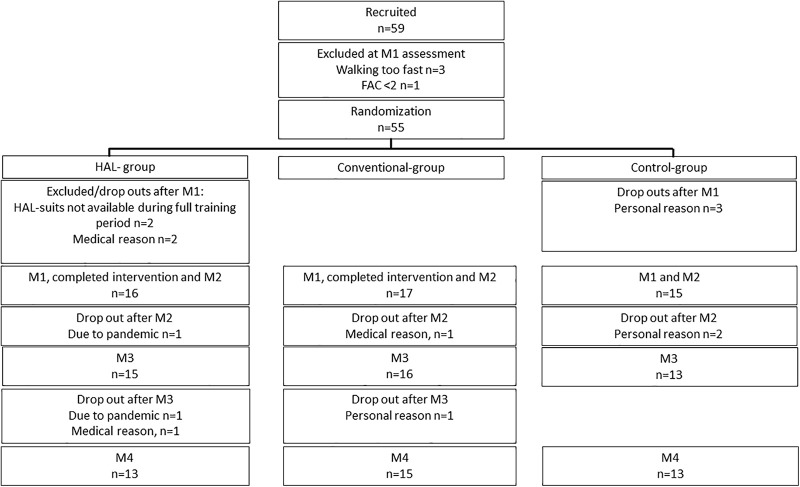
Participant flow diagram.

The first intervention period started in October 2015. The process of identifying eligible participants from outpatient care was slower than anticipated and the number of dropouts was higher than anticipated. Due to unforeseen logistic reasons related to transportation of the suits, amid a training period with HAL, two participants had to be excluded. The last participants were included in September 2019. The pandemic in 2020 prevented 1 follow up at 6 months and 2 at 12 months. Included participants and follow ups are presented in Figure 2.

### Adverse Events

Adverse events included occasional transient redness or abrasion of the skin and discomfort or pain, related to pressure from the HAL-suit, the attached shoes or electrodes (*n* = 6). One participant in the conventional group experienced transient pain in the ankle after a fall.

### Participant Characteristics

Participant characteristics including overall level of functioning is presented in Table 1.

**TABLE 1:** Characteristics of participants who completed the M2 assessment.

	HAL-group	Conventional group	Control group
	*n* = 16	*n* = 17	*n* = 15
Age, years mean (SD)	62.25 (7.90)	61.65 (8.59)	60.00 (7.29)
Female/Male	5/11	6/11	2/13
Housing, cohabitant/single/24 h assistance	11/5/0	9/7/1	13/2/0
Diagnosis, hemorrhage/infarction/both	3/12/1	8/9/0	4/11/0
Time to inclusion, months, median (IQR)	21.00 (24.75)	38.00 (34.50)	28.00 (50.00)
Paretic side, right/left	6/10	4/13	6/9
National Institute of Health Stroke Scale, median (IQR)	7.00 (5.25)	8.00 (6.00)	8.00 (6.00)
Barthel Index, median (IQR)	80.00 (20.00)	85.00 (32.50)	80.00 (15.00)
Functional Ambulation Categories, median (IQR)	3.50 (2.00)	4.00 (1.50)	3.00 (2.00)
Modified Ranking Scale, median (IQR)	3.00 (1.00)	3.00 (1.00)	3.00 (1.00)

*Standard deviation (SD), Inter quartile range (IQR).*

### Training Time and Walking Distance During the Intervention Period

Number of training sessions, time and walking distance during the intervention period is presented in Table 2.

**TABLE 2:** Completed total number of scheduled training sessions and total scheduled time in all groups.

	HAL-group	Conventional group	HAL/Conv *p*-value	Control group	HAL/Conv/Contr *p*-value
Completed scheduled number of training sessions, mean (SD)	16.69 (2.39)	16.75 (2.23)	0.289	6.47 (4.36)*	0.000
Completed scheduled training time in minutes, mean (SD)	1503.75 (208.39)	1402.94 (301.97)	0.276	358.33 (255.59)*	0.000
Completed effective training time in minutes, mean (SD)	851.94 (325.60)	845.10 (251.12)	0.946	NA	NA
Completed walking distance in meters, mean (SD)	15420.13 (9811.13)	6323.29 (3840.20)	0.002	NA	NA

Total distance walked and total effective training time in the HAL-group and Conventional (Conv) group. Control group (Contr). *Reported in weekly telephone interview. Standard deviation (SD).

*Post-hoc* analysis showed a significantly lower number of completed training sessions and minutes in the Control group compared to the HAL and Conventional group, respectively (*p* < 0.001, *p* < 0.001 according to Tukey HSD and Bonferroni).

### Clinical Assessments at M1 and M2

Results of the clinical assessments at M1 and M2 are presented in Table 3.

**TABLE 3:** Results of the clinical assessments at M1 and M2 and test of significant differences (*p*-value) within groups (based on results at M1 and M2) and between groups (based on the change in results between M1 and M2).

Assessments	HAL M1	HAL M2	HAL M1-M2	Conv M1	Conv M2	Conv M1 -M2	HAL vs. Conv	Contr M1	Contr M2	Contr M1-M2	HAL vs. Conv
	*n* = 16	*n* = 16	*p*-value	*n* = 17	*n* = 17	*p*-value	*p*-value	textitn = 15	*n* = 15	*p*-value	vs.Contr
											M1, M2
											*p*-value
6 minute walk test, meter, mean (SD)	80.11 (51.98)	87.64 (51.73)	0.253	84.75 (50.74)	106.44 (58.92)	0.002	0.111	113.37 (80.73)	110.19 (79.37)	0.655	0.029
Borg RPE Median (IQR)	13.00 (1.75)	13.00 (5.25)	0.245	13.00 (2.00)	13.00 (2.50)	0.369	0.204	13.00 (4.00)	13.00 (4.00)	0.553	0.418
Berg Balance Scale, mean (SD)	33.75 (12.00)	36.44 (9.98)	0.072	34.94 (11.94)	37.71 (11.71)	0.000	0.963	31.93 (12.07)	34.13 (12.39)	0.082	0.936
FMA-LE, total score, mean (SD)	60.56 (12.13)	62.25 (9.64)	0.414	61.12 (8.67)	63.71 (8.12)	0.093	0.963	56.73 (12.20)	58.40 (9.95)	0.421	0.784
FMA-LE, motor score, mean (SD)	15.44 (5.76)	15.88 (6.11)	0.639	17.29 (4.83)	19.00 (4.44)	0.087	0.339	16.53 (7.60)	16.73 (7.03)	0.845	0.482
10 meter walk test, meter/second, mean (SD)	0.27 (0.16)	0.29 (0.14)	0.127	0.29 (0.16)	0.34 (0.18)	0.010	0.159	0.33 (0.22)	0.35 (0.24)	0.508	0.193
Stroke Impact Scale, mobility domain, mean, SD	63.71 (18.19)	74.86 (20.11)	0.025	67.36 (21.11)	74.91 (20.47)	0.036	0.737	66.11 (17.76)	66.11 (17.15)	1.000	0.134

*Conventional (Conv), Control (Contr), Fugl-Meyer Assessment—Lower Extremity (FMA-LE), Standard deviation (SD), Inter Quartile range (IQR).*

A significant difference between the three groups in 6 MWT change (M1 M2 Δ) was found (*p* = 0.029 using one way ANOVA). *Post-hoc* Test revealed a significant difference between the Conventional group and the Control group (*p* = 0.022 according to Tukey HSD and Bonferroni *p* = 0.025) but not between the HAL-group and Conventional group (*p* = 0.258 Tukey HSD) or the HAL-group and the Control group (*p* = 0.447 Tukey HSD). No other significant group differences were identified (Table 3).

### Factors Associated With Changes in Walking Distance

Including all three groups, a significant positive correlation was found between completed number of training sessions and changes in walking distance (Δ M1 M2, 6 MWT *r* 0.317 *p* = 0.028).

In the intervention groups, the total distance (meters) walked during the training sessions was not significantly correlated with changes in accomplished walking distance after the intervention (Δ 6 MWT) (both groups: *r* −0.155 *p* = 0.388, HAL-group: *r* −0.153 *p* = 0.572, Conventional group: *r* 0.368 *p* = 0.146).

In the HAL-group, participants with FAC 4 (*n* = 8) walked twice as far as participants with FAC 2 (*n* = 7) [meters in mean (SD), FAC 4: 19689. 49 (5863.50), FAC 2: 10982.50 (12315. 19)] during the training sessions. The remaining participant with FAC 3 walked 12328.60 m. In the Conventional group participants with an FAC 4 (*n* = 9) walked more than 3 times longer distance than participants with FAC 2 (*n* = 4) [mean (SD) FAC 4: 9366.00 (1682.79), FAC 2: 2595.75 (2104.44)] and FAC 3 (*n* = 4) walked in mean 3204.75 m (SD 2720.99). However, distance walked within the FAC categories did not significantly change the outcome (Δ M1 M2, 6 MWT) between the training groups (FAC 2 and 3, *p* = 0. 878, FAC 4 *p* = 0.059).

In the intervention groups, the accomplished total effective training time in minutes during the training period was not significantly correlated with changes in accomplished walking distance after the intervention (Δ 6 MWT) (both groups: *r* 0.148 *p* = 0.227, HAL-group: *r* -0.049 *p* = 0.856, Conventional group *r* 0.041 *p* = 0.094), but rather with initial walking ability [6 MWT at baseline (M1)] in the HAL-group although not in the Conventional group (both groups: *r* 0.605 *p* < 0.001, HAL group *r* 0.748 *p* = 0.001, Conventional group: *r* 0.439 *p* = 0.078, Pearson correlation 2-tailed).

No significant associations between changes in perceived mobility (SIS mobility domain) and changes in assessed walking distance were found (HAL-group: R square 0.049 *p* = 0.429, Conventional group: R square 0.018 *p* = 0.630).

### Longitudinal Changes in Accomplished Walking Distance

**FIGURE 3:**
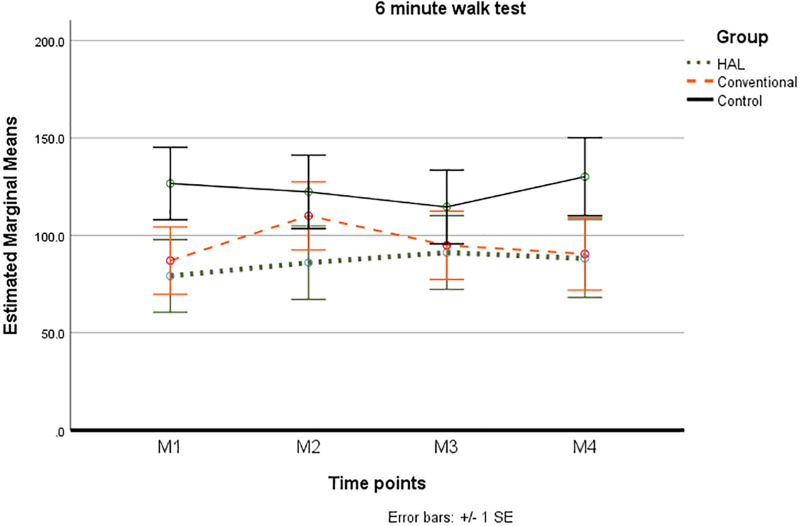
Six minute walk test, in meters, presented as estimated marginal means (±1 Standard deviation) at each time point M1 (baseline), M2 (post intervention), M3 (6 month follow up), M4 (12 months follow-up).

Linear mixed model was performed with 6 MWT as dependent variable, controlling for (1) group allocation or (2) time and a (3) group and time interaction (time ^∗^ group). Tests of fixed effects was significant for time^∗^group (*p* = 0.011). Based on estimated marginal means, pairwise comparisons showed significant improvements between M1 and M2 in the Conventional group (*p* = 0.006) and a significant decrease between M2 compared to M3 and M4, respectively (*p* = 0.043) (adjusted for multiple comparisons with Bonferroni). No significant changes were found in the HAL-group or Control group at any time point (Figure 3).

### The Effect of Predictive Variables on Longitudinal Changes in Accomplished Walking Distance

In the following linear mixed models, predictive variables were included in the model one at a time. Significant results are presented in Table 4.

**TABLE 4:** Significant results of the linear mixed models including the 6 Minute Walk Test as a dependent variable and controlling for group and time and group and time interaction.

Predictors (collected at M1)	Tests of fixed effects	Akaike’s Information Criterion (AIC)	Estimate
Berg Balance Scale	Predictor *p* < 0.001 Time*Group *p* = 0.014	1606.363	4.00
Modified Ranking Scale	Predictor *p* < 0.001 Time*Group *p* = 0.010	1616.134	68.58
Barthel Index	Predictor *p* < 0.001 Time*Group *p* = 0.012	1622.016	2.47
National Institute of Health Stroke Scale	Predictor *p* = 0.041 Time*Group *p* = 0.012	1638.694	-4.89

*A significant result was found for time and group interaction (Time^∗^Group) as presented in the table.*

The best fitting model included the Berg balance scale with an estimate of 4.00. Based on estimated marginal means significant improvements, were only found between M1 and M2 in the Conventional group (mean difference 21.69 *p* = 0.006) followed by a significant decrease between M2 compared to M3 (mean difference -15.38 *p* = 0.043) and M4, respectively (mean difference -19.53 *p* = 0.046) (adjusted for multiple comparisons with Bonferroni).

These significant changes based on estimated marginal means found between time points only in the Conventional group were repeated in all models with significant tests of fixed effects (Table 4).

## Discussion

This randomized controlled trial including ambulatory participants, living with long-term impairments and activity limitations after stroke, demonstrated improvements in walking distance in both intervention groups while the Control group declined. However, while the Conventional group improved significantly compared to the Control group, no significant difference was found between the HAL-group and Control group. Thus, while previous studies have found no, or significant improvements when comparing exoskeletal gait training to conventional training (Mehrholz et al., 2017b; Bruni et al., 2018; Tedla et al., 2019), results from the present study indicate that intensive gait training with the HAL exoskeleton where the distance walked was even doubled compared to the Conventional group, is no better than the low intensity training reported in the Control group with regard to the primary outcome of this study. These results may be surprising as high intensity task-specific training is the core of evidence-based training after stroke (Langhorne et al., 2011) and several factors may probably play a role.

Factors that need to be taken into consideration are that HAL-training was performed on a treadmill to enable safe walking with stationary BWS and that the HAL suit is limited to motions only in the sagittal plane. Treadmill training has been found to improve walking endurance and cardiovascular fitness long-term after stroke but among participants with a higher level of functioning than the participants in the current study (Globas et al., 2012). A Cochrane review including studies on treadmill training with body-weight support (Mehrholz et al., 2017a) found that patients who were dependent in walking did not increase their walking endurance after this type of training. These results are compatible with the results of the current study where a longer walking distance at baseline was not correlated with improvements in accomplished walking distance (6MWT) after the intervention. It appears that adding an exoskeleton does not improve these results. One may even speculate if the use of a treadmill and BWS may have negatively affected outcome in the HAL-group. While use of a treadmill and BWS may enable high intensity gait training it may not be sufficiently task-specific to generalize to over ground walking and walking in an everyday life environment (Langhorne et al., 2011; Mehrholz et al., 2017a). However, it should be pointed out, that other outcomes such as cardiovascular risk factors, not reported here, may improve in response to long walking distances. Further, the current results are not valid for other training procedures with HAL like over ground walking and current developments in the exoskeleton area may offer further possibilities. During the last decade, there has been remarkable progress in development of exoskeletons for walking while taking on the challenge of creating devices adjustable and usable during multiple degrees of freedom, and to different activities and tasks. Textile exoskeletons are one example, with less kinematic restrictions and improved usability (Awad et al., 2020; Sawicki et al., 2020).

Participants in the current study represent a subgroup of relatively young persons, with remaining impairments and partially dependent in walking in the long-term stage after stroke. Thus, the results cannot be generalized to a majority of the stroke population. However, the current subgroup constitutes approximately 15% of the total stroke population (<75 years) in Sweden who do not reach full independence (The Swedish Stroke Register Riksstroke, 2018) in mobility after stroke. In addition to the drastic negative long-term effects on activity and participation and economic consequences due to productivity loss for the individual, approximately 40% of the total economic cost for society have been found to fall on rehabilitation and long-time social services and informal care during the first year after stroke (Persson et al., 2012). Moreover, up to 45% of the costs of stroke have been found to be attributed to social services long-term (Ghatnekar et al., 2004). Thus, although this subgroup is relatively small, the individual and societal burden is vast and interventions aiming to increase their level of independence are warranted.

The results of this study agree with previous studies indicating that intensive conventional training may have beneficial effects in ambulatory persons living with long-term impairments and activity limitations after stroke (Hornby et al., 2020). However, since improvements observed at the end of the intervention period did not reach a meaningful clinical important difference (Tang et al., 2012) in the conventional training group and did not remain at the 6 and 12 months follow up, there is a need for further studies to elucidate if improvements observed here were not enough to alter everyday activity patterns that would promote sustained functioning and if the rehabilitation period was too short.

The current study focused on gait training combined with balance, strength and task specific mobility training, followed by a home visit to identify activities in daily living where the participant could make use of any gains in functioning and enhance sustainability. Interestingly, although significant improvements in functioning was only identified in the Conventional group, both intervention groups reported a significant improvement in perceived mobility in activities of daily living that may be interpreted as a clinically meaningful difference (Lin et al., 2010). However, these improvements could not be explained by gains in assessed functioning. Other factors that may be involved will be explored in a forthcoming study based on semi structured interviews with the participants in the HAL-group. Further studies should focus even more on integrating gait training in everyday activities in the home setting to achieve a level of functioning where the improvements enhance independence and participation and become sustainable.

Further studies may also consider the predictors affecting outcome in the Conventional group observed in this study, i.e., overall stroke severity, balance, independence in personal care and mobility as well as overall activity performance and participation. These factors also affected the decline over time seen after the intervention. Thus, focus on improved balance and independence in walking as well as the transfer package to prevent deterioration may be recommended as well as longer duration of the intervention period and integration of training tasks in everyday activities. Other factors to consider include cognitive function such as the influence of executive and visuospatial function on functional outcome and sustainability. These factors will be explored and reported on in a separate publication.

The turning point during 6MWT, introduced due to construction work in the building where testing took place, may have affected the results, although most likely only to a minimal extent. Importantly, any such effect would then presumable have been similarly distributed between groups given the block randomization. Another issue in the current study is power. Due to the number of drop-outs in the Control group, only 15 of the 16 planned participants could be reassessed after M2. However, data from this study allow some conclusions and may guide the design of further studies.

## Conclusion

This study demonstrates that 6 weeks of intensive, conventional training focused on walking may significantly improve walking when performed by patients, who are ambulatory but dependent in walking, in the long-term phase after stroke. In contrast, EAGT on a treadmill, which allowed significantly longer walking distances during training, had no significant additional effect on walking. The results of this study may guide clinicians, managers and stake holders in providing cost effective evidence-based interventions for gait training long-term after stroke and also suggest areas for further studies.

## Data Availability Statement

The raw data supporting the conclusions of this article will be made available by the authors, without undue reservation, to any qualified researcher.

## Ethics Statement

The studies involving human participants were reviewed and approved by the Swedish Ethical Review Authority, a Swedish state agency. The patients/participants provided their written informed consent to participate in this study. Written informed consent was obtained from the individual(s) for the publication of any potentially identifiable images or data included in this article.

## Author Contributions

JB, SP, and AW have initiated and made substantial contribution to the conception and design of the study and in writing the manuscript. PL, KV, AD, KS, and CH have contributed to the design and revised the manuscript critically for important intellectual content. All authors contributed to the article and approved the submitted version.

## Conflict of Interest

The authors declare that the research was conducted in the absence of any commercial or financial relationships that could be construed as a potential conflict of interest.
